# Physical human locomotion prediction using manifold regularization

**DOI:** 10.7717/peerj-cs.1105

**Published:** 2022-10-12

**Authors:** Madiha Javeed, Mohammad Shorfuzzaman, Nawal Alsufyani, Samia Allaoua Chelloug, Ahmad Jalal, Jeongmin Park

**Affiliations:** 1 Department of Computer Science, Air University, Islamabad, ICT, Pakistan; 2 Department of Computer Science, College of Computers and Information Technology, Taif University, Taif, Saudi Arabia; 3 Department of Information Technology, College of Computer and Information Sciences, Princess Nourah bint Abdulrahman University, Riyadh, Saudi Arabia; 4 Department of Computer Engineering, Tech University of Korea, Sangidaehak-ro, Gyeonggi-do, South Korea

**Keywords:** Features optimization, Human motion analysis, Physical motion classification, Patterns decision, Manifold regularization, Inertial signal filter

## Abstract

Human locomotion is an imperative topic to be conversed among researchers. Predicting the human motion using multiple techniques and algorithms has always been a motivating subject matter. For this, different methods have shown the ability of recognizing simple motion patterns. However, predicting the dynamics for complex locomotion patterns is still immature. Therefore, this article proposes unique methods including the calibration-based filter algorithm and kinematic-static patterns identification for predicting those complex activities from fused signals. Different types of signals are extracted from benchmarked datasets and pre-processed using a novel calibration-based filter for inertial signals along with a Bessel filter for physiological signals. Next, sliding overlapped windows are utilized to get motion patterns defined over time. Then, polynomial probability distribution is suggested to decide the motion patterns natures. For features extraction based kinematic-static patterns, time and probability domain features are extracted over physical action dataset (PAD) and growing old together validation (GOTOV) dataset. Further, the features are optimized using quadratic discriminant analysis and orthogonal fuzzy neighborhood discriminant analysis techniques. Manifold regularization algorithms have also been applied to assess the performance of proposed prediction system. For the physical action dataset, we achieved an accuracy rate of 82.50% for patterned signals. While, the GOTOV dataset, we achieved an accuracy rate of 81.90%. As a result, the proposed system outdid when compared to the other state-of-the-art models in literature.

## Introduction

Predicting the human activities performed can also be referred to as human dynamics analysis and prediction (Xiao et al., 2012). The term locomotion detection is relatively novel as compared to motion detection. It is used in different capacities including locomotion prediction for estimating walking targets and redirected walking (Zank & Kunz, 2015, 2017), locomotion prediction in virtual reality for tracking space while walking (Stein, Bremer & Lappe, 2022; Bremer, Stein & Lappe, 2021), and locomotion recognition *via* human actions recognized (Yan et al., 2018; Ghadi et al., 2022). In the proposed system, our goal is to predict the human locomotion *via* multiple physical activities detection.

Some researchers proposed human locomotion analysis and prediction using motion sensors (Jalal, Quaid & Hasan, 2018; Haneche, Ouahabi & Boudraa, 2019), while others preferred vision-based sensors (Jalal et al., 2021; Gochoo et al., 2021). The applications of human locomotion prediction systems include smart home environments (Jalal et al., 2017), security and healthcare systems (Jalal, Kim & Kim, 2014), behavior mining (Jalal & Mahmood, 2019), life logging systems (Jalal, Kim & Kim, 2012), and smart surveillance (Pervaiz, Jalal & Kim, 2021). For such real-world applications (Quaid & Jalal, 2020) a variety of sensors are utilized and, the methods are still lacking in human motion prediction with minimal circumstantial information. The above-mentioned applications require recognition of both simple and complex motion patterns that is missing in literature. Due to the motion containing intervals, delays, jerks *etc.,* it becomes difficult for the system to predict human locomotion dynamics. Therefore, considering the need, we refer simple motion patterns as static and complex motion patterns as kinematic in this research.

We propose a novel technique for the prediction of human motion (PHM) using kinematic-static patterns identification. Data is obtained from two benchmark datasets, namely, PAD (Khan et al., 2020) and GOTOV (Paraschiakos et al., 2020). For noise reduction in the signals acquired, this article develops a calibration-based filter for inertial measurement unit (IMU) data. The filter helps in reducing the errors present in the raw IMU signals. A Bessel filter is applied for the physiological data from sensory devices like electromyography (EMG) and electrocardiography (ECG). By applying these filters, we got clean data to perform further processing and achieve better results. Next, this filtered data from IMU and physiological sensors is fused together over time. Then, the data is divided into windows of 2 s each for detailed analysis of the fused signals.

Next, to decide the kinematic-static patterns among the windows (Al Shloul et al., 2022), we suggested a polynomial probability distribution. For kinematic patterned signal data, multisynchrosqueezing transform (Yu, Wang & Zhao, 2019) and hidden Markov random field (Wang, 2012) are suggested for features extraction, whereas, for static patterned signal windows, dynamic time warping (Laperre, Amaya & Lapenta, 2020) and Gaussian Markov random field (Khalid et al., 2021) are recommended. Then, two feature optimization techniques are used including quadratic discriminant analysis and orthogonal fuzzy neighborhood discriminant analysis. Lastly, manifold regularization using multiple algorithms is applied to evaluate the performance of our proposed PHM model.

The key contributions of this research are:

- Our PHM model is a novel technique of pre-extraction for kinematic and static patterned signals in order to extract and classify the complex motion patterns.
- For inertial human motion data, we designed a calibration-based filter that provides improved regulated and converged filtered data.
- For the prediction of indoor-outdoor activities, this article recommends a variety of features extraction approaches for each kinematic and static motion patterned data.
- Finally, two optimization techniques including orthogonal fuzzy neighborhood discriminant analysis along with quadratic discriminant analysis and manifold regularization algorithms provide better comparison of the proposed PHM model.

The article is divided into sections as: it provides the related work section followed by the material and methods section illustrating the overview of our proposed technique and detailed study in the noise reduction, data fusion and windowing, kinematic-static patterns decision, features extraction, features optimization, and classification using manifold regularization sub-sections. Then, the results section presents experiments conducted and outcomes of the system and finally, we concluded the article with an overall synopsis.

## Related work

A variety of advanced human motion analysis approaches have been studied and utilized in indoor-outdoor physical monitoring that can be further divided into motion-based and vision-based human dynamics prediction models. Motion sensors including accelerometer (Acc), gyroscope (Gyro), magnetometer (Mag), mechanomyography (MMG), ECG, EMG, and geomagnetic (GeoMag) are used by a variety of researches. Whereas, for vision-based systems, Microsoft Kinect RGB-D cameras (Kinect), Intel Realsense (Realsense), Asus Xtion (Xtion), video cameras (video cam), and dynamic vision sensor (DVS) are utilized. Table 1 presents a literature review for human dynamics prediction *via* motion sensors and vision sensors based on recent studies.

**Table 1:** Literature review for existing PHM models.

Human dynamics prediction *via* motion sensors			
State-of-the-art models	Sensors details	Main contributions	Limitations
Jalal, Quaid & Kim (2019)	Acc	An accelerometer-based motion detection methodology is proposed using multi-features and random forest for classification. The system produced features including variance, positive-negative peaks, and signal magnitude features.	Although the model achieved good accuracy, it considered limited static activities such as drink glass, and pour water.
Chen et al. (2020)	Acc Gyro Mag ECG	A pattern-balanced semi-supervised deep model is proposed for imbalanced activity recognition from multimodal sensors. The study focused on multimodal sensors, limited labeled data and class-imbalance issues. Further, it has exploited the independence of multiple sensors based data and to identify salient regions that recognize human activities.	Imbalanced data distribution is a challenge, which authors tried to void. However, the system performance was low when compared to other methods.
Batool, Jalal & Kim (2019)	Acc Gyro	Method to recognize physical activity detection is proposed *via* features extraction like Mel-frequency cepstral coefficients (MFCCs). Further, particle swarm optimization and support vector machines (SVM) is used for classification.	Limited motion activities are recognized using Motion-Sense dataset, which will not fit over dynamic activities.
Javeed, Jalal & Kim (2021)	IMU MMG EMG	An effective model for healthcare monitoring has been proposed using multiple features, feature reduction, and recognizer engine. A novel multi-layer sequential forward selection technique has been proposed along with bagged random forest for classification.	The system recognized limited exercise-based activities but was unable to attain good accuracy rates.
Jalal et al. (2020)	Acc Gyro Mag	A detailed study on the physical activities detection systems has been presented in this research. Further, a quality of life improving method has been proposed for indoor-outdoor environments. Both statistical and non-statistical features extraction methods have been fused together to recognize multiple physical activity patterns.	Although the model achieved good accuracy, it recognized only static activities including downstairs, upstairs, and walking.
Xia et al. (2021)	Acc Gyro	The research presents twofold contributions towards sensor-based human activity recognition. First, it proposed a skinned multi-person linear model to build a large dataset based on forward kinematics. Second, it presented a novel deep learning model named multiple level domain adaptive learning model to learn the disentangled representation for the multi-sensors-based data.	The system was able to achieve acceptable rates but due to all the activities mixed together, the performance attained was not up-to-the-mark.
Azmat & Jalal (2021)	Acc Gyro GeoMag	The paper proposed a combination of template matching and codebook generation to eliminate the orientation errors and lessen the computational complexity. The overall methodology involves pre-processing, windowing, segmentation, features extraction, and classification techniques.	Method proposed template matching for static and dynamic activities, however, accuracy achieved for dynamic activities was low.
Ayman, Attalah & Shaban (2019)	Acc Gyro Mag	The paper proposed a novel framework for human activity recognition using machine learning based sensors fusion technique. It also utilized random forest, bagged decision tree, and SVM classifiers for the features selection. The proposed framework consists of data collection, segmentation, features extraction, and classification along with features selection methods.	Limited gestures have been predicted using Handy and PAMAP2 datasets, which will not be able to perform acceptable over dynamic activities.
Jalal et al. (2020)	Acc Gyro Mag	A combination of multiple sensors like accelerometer, gyroscope, and magnetometer have been used to recognize physical activities. Multiple types of features including statistical, MFCCs, and Gaussian mixture model have been extracted followed by the classification of multiple activities *via* decision tree.	Imbalanced data distribution is eluded. However, the system performance was very low when compared to other state-of-the-art methodologies.
Tao et al. (2021)	Acc Gyro Mag	They proposed a novel attention-based approach for human activity recognition. First, they extracted sensor-wise features using convolutional neural networks (CNN). Then, they used attention-based fusion method for learning body locations and generating features representations. Lastly, inter-sensor features extraction has been applied to learn inter-sensor correlations and predict activities.	The model was able to achieve acceptable rates but due to all the activities mixed together, the performance accomplished was not decent enough.
Javeed et al. (2020)	Acc Gyro ECG EMG	Hybrid-features based sustainable physical healthcare patterns recognition (HF-SPHR) has been proposed in this research. The system includes pre-processing, features extraction, features fusion and reduction, codebook generation, and classification using deep belief networks.	Limited motion activities have been detected *via* selected datasets that is not sufficient to accomplish well over dynamic activities.

Human Dynamics Prediction *via* Vision Sensors			
State-of-the-art models	Sensors details	Main contributions	Limitations
Khaire, Kumar & Imran (2018)	RGB-D	The authors proposed a ConvNets based activity recognition using skeleton images, joint sequences, and RGB images. ConvNets are separately trained for each type of images and softmax scores are further fused at the decision level.	Although the proposed system achieved good results but vision-based datasets can cause privacy related issues.
Mahmood, Jalal & Kim (2020)	Video Cam	The WHITE STAG model was proposed to track human interactions using full-body silhouettes and skeleton joints. Linear discriminant analysis has been used for features reduction and kernel sliding perceptron has been utilized to recognize multiple human interaction classes.	Limited activities are detected *via* IM-IntensityInteractive7 dataset that is not sufficient for dynamic activities.
Pansuriya et al. (2020)	DVS	They used event-based dynamic vision sensors for human activity recognition. The steps include pre-processing, features extraction using CNN, and sequence learning *via* deep recurrent neural network (RNN) for human activity recognition.	Although the proposed method attained decent results but there are confidentiality problems.
Nadeem, Jalal & Kim, 2020	Cam	Approach for human action detection is proposed using artificial neural networks (ANN). Multi-dimensional features are extracted from twelve body parts. Multiple classifiers are utilized to compare the performance of the system.	The proposed model utilized a classifier with limited performance due to its rudimentary nature.
Baradel et al. (2018)	Cam Kinect	Without depending on pose information, the proposed system has been able to recognize human activities. Glimpses have been separated from recurrent actions and a visual attention model has been used to learn these glimpse sequences. Each worker has been assigned a glimpse.	Although the system achieved good accuracy rates, however, it has disputes with occlusions during human interactions.
Rafique, Jalal & Kim (2020)	Kinect RealSense Xtion	They proposed a method to understand and recognize human interaction scenes *via* depth data. Statistical multi-object segmentation is also used to learn scenes and separate different objects. Features are extracted from the separated objects and a multilayer perceptron has been suggested for whole scene recognition including humans.	This videos-based system requires continuous monitoring and to generate reliable answers at the right time, which is difficult to achieve in real-time systems.
Alrashdi et al. (2021)	Kinect	A maximum entropy Markov model (MEMM) based approach is proposed. A modified Viterbi algorithm to generate the most similar sequence based on observations is proposed. A depth dataset has been used to evaluate the performance of the system.	Depth-based datasets cannot work without depth maps, which consumes a lot of memory and time.
Ghadi et al. (2021)	Video Cam	A novel technique is proposed to study the human posture and gait event detection. Starting with posture and landmark information extraction and 2D skeleton mesh extraction, human 2D to 3D model has been reconstructed. A rule-based features mining technique has been utilized and deep-learning model CNN has been used for human landmarks detection.	Limited activities have been detected *via* mpii-video pose and COCO datasets that has limited dynamic recognition due to complexity in videos and group density.
Saini et al. (2018)	Kinect	They presented a framework for continuous human activity recognition by capturing activity sequences as 3D skeleton trajectories consisting of 3D positions of different joints estimated. Bi-directional long short-term memory neural networks have been used to classify these activities.	Depth-based datasets cannot recognize activities without depth maps, which consumes a lot of memory and time.

From Table 1, it is observed that predicting complex human activities is still a challenge. Besides, relevant datasets need to be used with more variety of human motion. More importantly, the prediction time, complexity, and accuracy are three parameters that should be considered while recognizing human activities in PHM systems.

## Materials and Methods

**Figure 1:**
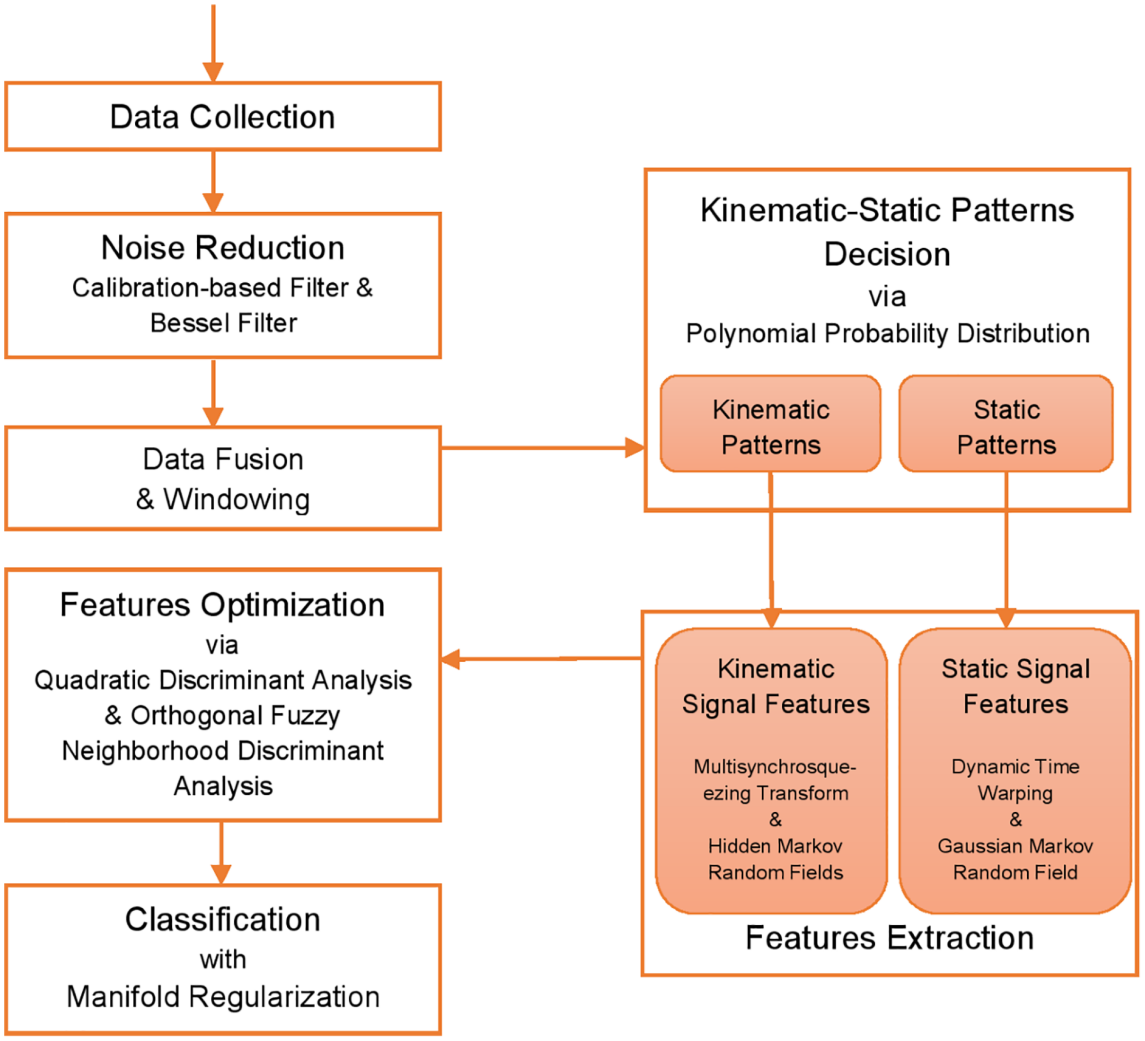
Flow chart illustrating the proposed PHM model using PAD Khan et al. (2020) and GOTOV Paraschiakos et al. (2020) datasets.

Data is collected from two publicly available datasets, namely, PAD and GOTOV. Figure 1 demonstrates the flow diagram of the proposed PHM model. After data collection, noise reduction techniques are applied followed by data fusion and extracting overlapping sliding windows of 2 s each. Next, the windows are used to decide for the kinematic and static patterns definition, which are further used to extract features. Then, the features are optimized using two methods named quadratic discriminant analysis (QDA) and orthogonal fuzzy neighborhood discriminant analysis (OFNDA). Finally, manifold regularization is used to verify the performance of the proposed system.

### Noise reduction

Pre-processing is required on the raw data in order to reduce the noise, biasness, and other errors in the IMU and other physiological signals acquired. For this, we have proposed a calibration-based filter for IMU data and Bessel filter for other physiological data.

#### Calibration-based filter for IMU

A three-phased calibration-based filter is proposed for this PHM model. This filter takes care of all the noise present in the IMU signals as it has three phases for denoising the signal. It has a calibration phase, where all the three types of IMU signals including accelerometer, gyroscope, and magnetometer are calibrated and filtered for noise. Next, during the error correction phase, earth’s gravitational field is utilized to reduce errors in acceleration.

**Figure 2:**
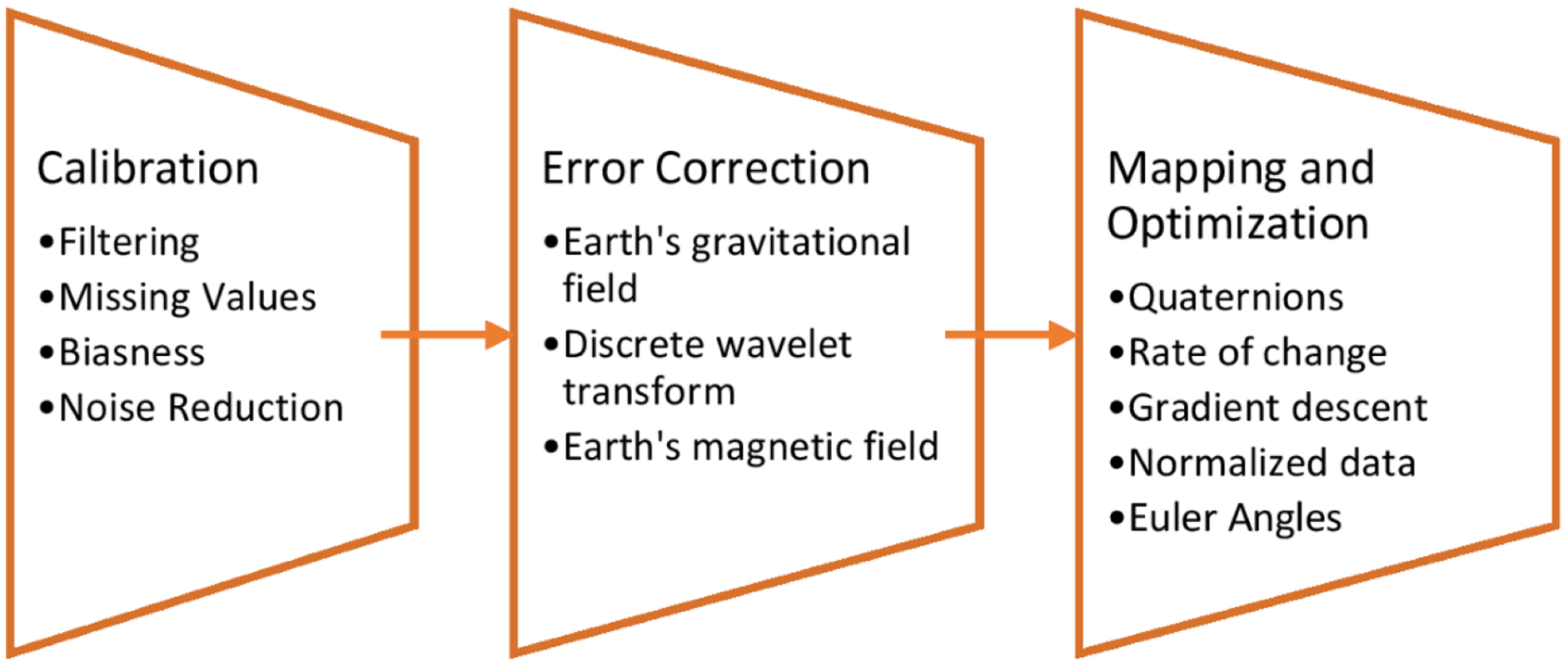
Three phased calibration-based IMU filter.

For gyroscope signals, a discrete wavelet transform technique is used to remove errors, whereas for the magnetometer signals, we used the earth’s magnetic field. Finally, in the final phase of mapping and optimization, the article proposes to map the error-corrected gyroscope signals to Quaternions. An optimal solution for the rate of change in drift is defined by gradient descent technique (Baldi, 1995). Figure 2 shows the brief description of each phase for the calibration-based filter over IMU sensor data.

#### Bessel filter for physiological sensors

It is a type of linear filters that provides maximum flat phase delay. Also, it keeps the shape of the wave near to original (Soni & Gupta, 2020). For physiological signals, we prefer to use a filter that will not change the characteristics of the signal itself. Therefore, we applied a Bessel filter that includes use of a transfer function as described in Eq. (1):

(1) $${\rm H}\left( {\rm t} \right) = \; \displaystyle{{{\theta _n}\left( 0 \right)} \over {{\theta _n}\left( {\displaystyle{t \over {{\omega _0}}}} \right)}}$$

where ${\theta _n}\left( t \right)$ is a reverse Bessel polynomial and ${\omega _0}$ is the cut-off frequency.

### Data fusion and windowing

After calibration and filtering signals using two types of filtration methods, the PHM model proposes to fuse the two types of signals together. For data fusion, we suggest the time-based fusion that will combine the two types of filtered data together based on time.

Furthermore, sliding overlapping windows of 2 s each have been applied to take chunks of continuous signals and retrieve the different activities’ characteristics. Algorithm 1 shows the detailed procedure of data fusion and windowing over both IMU and physiological signals.

**Algorithm 1:** Sensors data fusion & windowing

**Input:** IMU: inertial measurement unit signals
PHY: physiological signals
**Output**: FUS: fused data signals
winSig: windowed signal
/* IMU has the calibrated and fused data from calibration-based IMU filter*/
/* T is for total time*/
/*totalVal is for total data*/
/* m is for number of total windows*/
**Step 1:**
**Repeat**
FUS(T) = IMU(T) U PHY(T);
**Until** end of T.
**Step 2:**
pVal = totalVal/T; /*per second data*/
pVal = pVal*2; /*two seconds data*/
**Repeat**
s = 1; /*window starting point*/
** For** i=1 to m **do**
** For** i=s to pVal **do**
winSig = FUS(i);
**End**
s = s+pVal;
**End**
**Until** end of Fused data achieved.

### Kinematic-static patterns decision

The unique method to decide about kinematic or static patterns has been introduced in order to facilitate the PHM model for prediction.

#### Polynomial probability distribution

**Figure 3:**
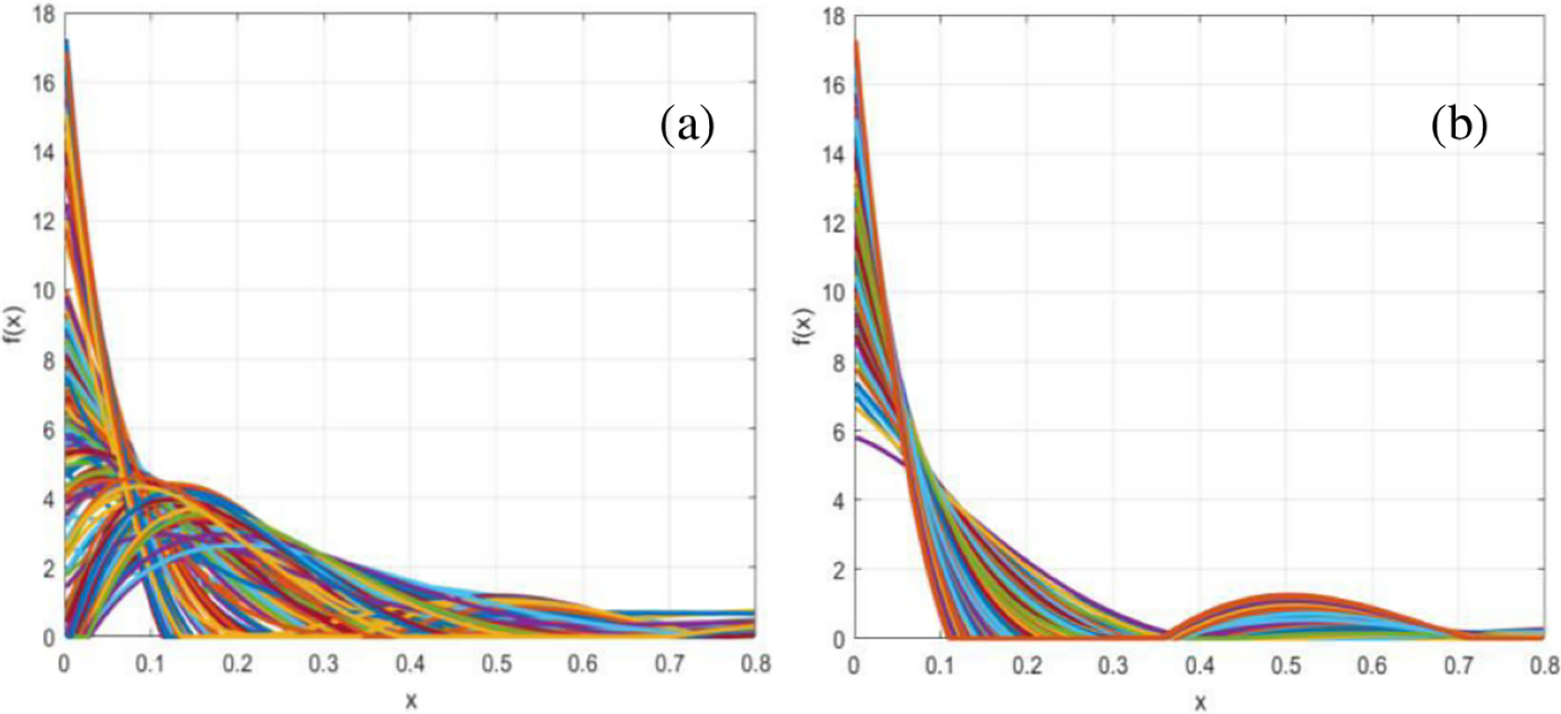
Polynomial density function distribution for (A) kinematic patterns and (B) static patterns.

Probability density function (PDF) gives the likelihood of an outcome in a given sample space and provides an acceptable difference between both kinematic and static patterns. A goodness of fit test is defined for the PDF approximation followed by polynomial degree selection and a real valued interval is defined as $\left[ {x,\; y} \right]$ (Munkhammar, Mattsson & Ryden, 2017). Equation (2) shows the PDF defined as ${M^{th}}$ order polynomial $P$ on the interval $\left[ {x,\; y} \right]$:

(2) $$P\left( {M,a} \right) = {w_0} + \; {w_1}a + \; {w_2}{a^2} + \ldots + \; {w_M}{a^M}$$

where ${w_m}$ is the unknown weight for each $m\; \in [ {0, \ldots ,M} ]$. Figure 3 represents polynomial density function comparison for kinematic and static patterns.

### Features extraction

After the patterns decision for kinematic and static, the PHM model suggest to extract features for both pattern types separately. Due to the kinematic patterns being more complex and variable in nature, dynamic time warping (DTW) and Gaussian Markov random field (GMRF) are selected for the features extraction from kinematic signals. Whereas, multisynchrosqueezing transform (MSST) and hidden Markov random field (HMRF) are applied over the static signals for features extraction as they tend to have pause and delays in them.

#### Dynamic time warping for kinematic patterns

For kinematic features extraction, DTW is used as one of the techniques. This technique is not limited in its application and has been used in multiple areas like speech (Amin & Mahmood, 2008), biology (Petitjean et al., 2014), economics (Franses & Wiemann, 2020) *etc*. It is used for the time-based comparison between two signals even if the signals are stretched or shifted in time (Laperre, Amaya & Lapenta, 2020). Therefore, DTW provides good results for IMU signals. If we take two time windows $P$ and $R$, then we can represent them as shown in Eqs. (3) and (4):

(3) $$P = \left[ {{p_1},\; {p_2},\; \ldots ,\; {p_i},\; \ldots ,\; {p_m}} \right]$$

(4) $$R = \left[ {{r_1},\; {r_2},\; \ldots ,\; {r_j},\; \ldots ,\; {r_n}} \right]$$

**Figure 4:**
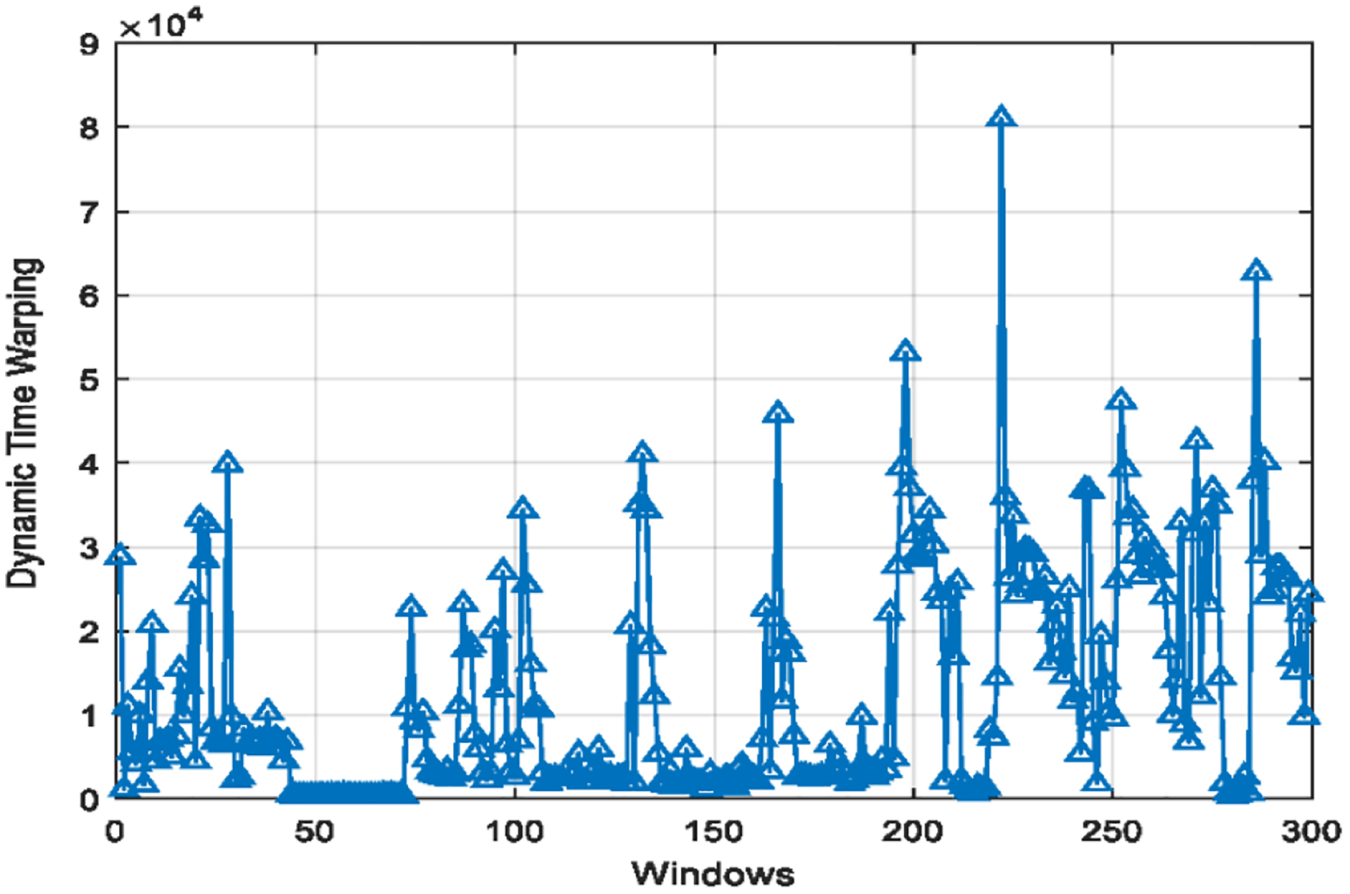
Dynamic time warping for kinematic patterned signals.

DTW firstly calculates the distance between $P$ and $R$ using Euclidean distance formula as in Eq. (5). Then, it searches for the warping path $PT$ as shown in Eq. (6):

(5) $$d\left( {p,r} \right) = \; \sqrt {{{\left( {p - r} \right)}^2}}$$

(6) $$PT = [p{t_1},\; p{t_2},\; \ldots ,\; p{t_k}]\; {\rm with} \;\max \;\left( {m,n} \right) \le k\; < m + n - 1$$

where each $p{t_k}$ represents the grid made using ${p_m}$ and $\; {r_n}$. Equation (7) shows the formula to calculate DTW as the cost function. Figure 4 displays the DTW calculated for two types of kinematic patterned window signals including motion and physiological.

(7) $$DTW\left( {P,R} \right) = \min \sqrt {\mathop \sum \nolimits_{i = 1}^k p{t_k}}$$

#### Gaussian Markov random field for kinematic patterns

**Figure 5:**
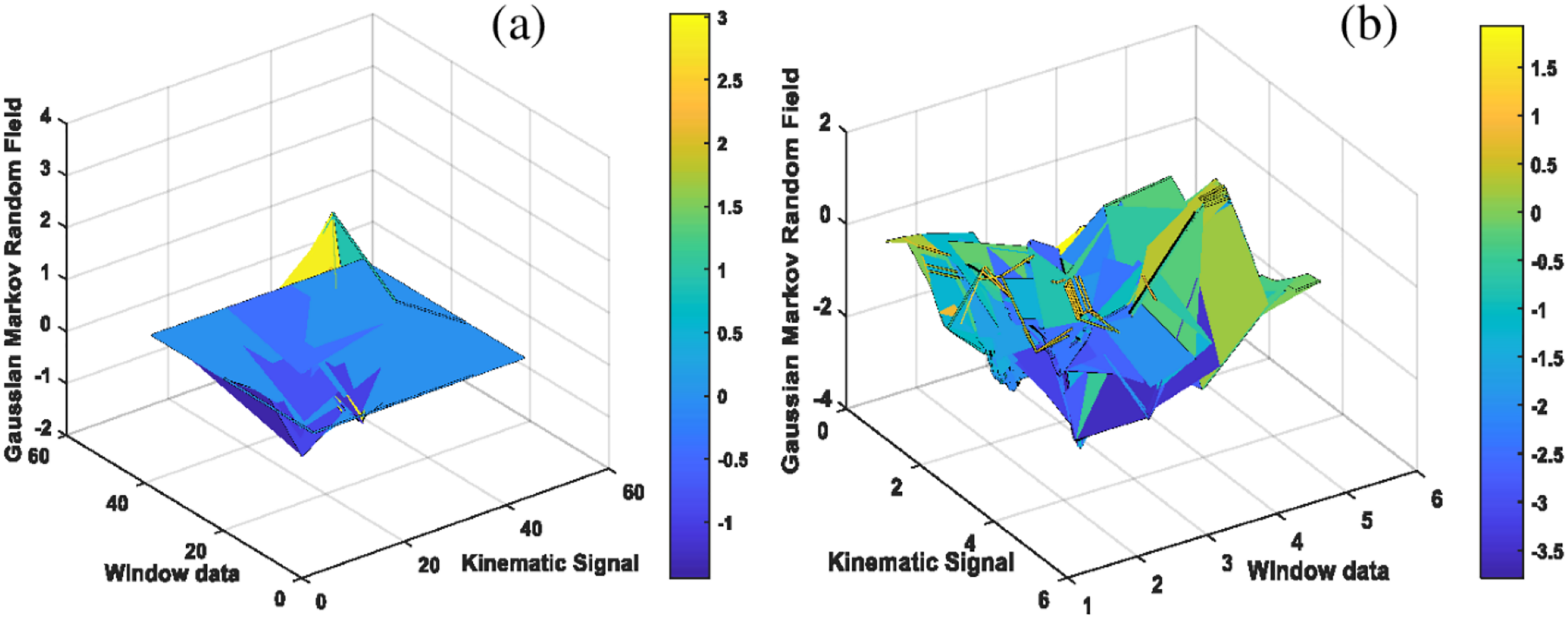
The results of Kinematic Gaussian Markov random field for complex motion patterns including (A) lifting heavy objects and (B) pushups.

Random fields is the general form of stochastic process where we don’t need the real values and it can take multi-dimensional matrix or points Khalid et al. (2021). Hence, we applied it over multi-dimensional signals like motion and physiological signals together. A stochastic process becomes Gaussian when all its distributions are Gaussian normalized. To determine a Gaussian process, we need to determine its expectation function as Eq. (8) and covariance function as Eq. (9) using $s$ samples and $t$ times (Kreose & Botev, 2015). Figure 5 presents the results of two complex kinematic motion patterns using Gaussian Markov random field features.

(8) $${\tilde \mu _t} = { E}{\tilde X_t}$$

(9) $$\widetilde {\sum s,t} = { cov}\left( {{{\tilde X}_s},\; {{\tilde X}_t}} \right)$$

#### Multisynchrosqueezing transform for static patterns

**Figure 6:**
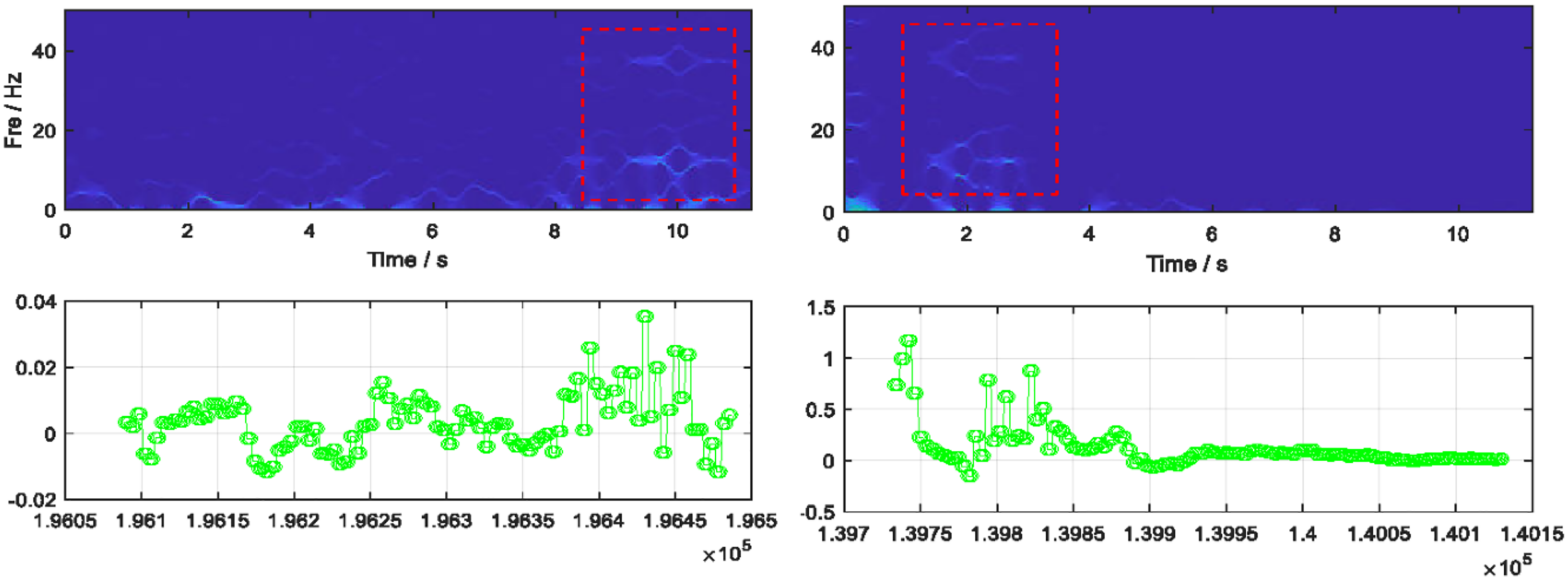
Multisynchrosqueezing transform features extracted for two random static patterned windows.

Synchrosqueezing transform (SST) assumes the signal under consideration to be weakly time varying and with time the SST representation becomes blurry. Therefore, an SST operation is required to be executed on already acquired SST results (Yu, Wang & Zhao, 2019). As the static patterned signals can be weak and become blurry over time, so we utilized the multiple iterative SST operations for the static signals resulting in Eq. (10) as MSST:

(10) $$T{s^{\left[ M \right]}}\left( {t,\gamma } \right) = \; \mathop \int \nolimits_{ - \infty }^{ + \infty } T{s^{M - 1}}\left( {t,\gamma } \right)\delta \left( {\gamma - \hat \omega \left( {t,\omega } \right)} \right)d\omega$$

where $M$ is the iteration number $\le$2 and $T{s^{\left[ M \right]}}\left( {t,\gamma } \right)$ represents the spread time-frequency coefficient. Figure 6 illustrates the MSST features extracted for random static windows.

#### Hidden markov random field for static patterns

**Figure 7:**
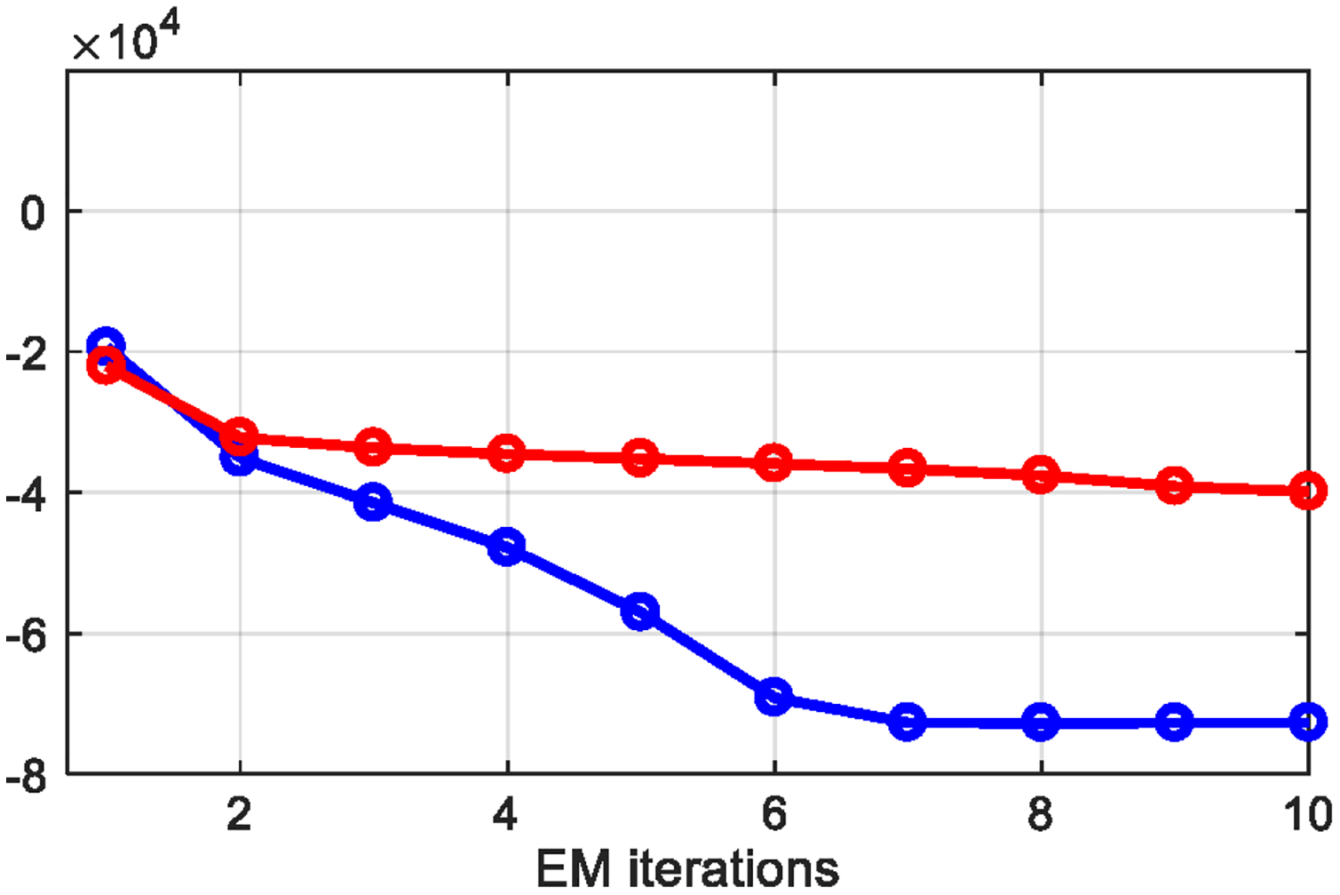
Prior energy extracted from each EM iteration.

The hidden Markov random field (HMRF) is applied (Wang, 2012), where the joint likelihood probability is described in Eq. (11).

(11) $$P\left( {y|x,{\Theta}} \right) = \; \prod  {_{i}} P({y_i}|{x_i},{\theta _{{x_i}}})$$

where $P({y_i}|{x_i},{\theta _{{x_i}}})$ denotes the Gaussian distribution and ${\Theta}$ shows the parameter set. To estimate the labels, MAP estimation is used by applying ${\Theta}$ parameter set. The prior energy function is used for MAP estimation as given in Eq. (12):

(12) $$U\left( x \right) = {\sum}_{c\; \varepsilon \; Cl} {F_c}\left( x \right)$$

where ${F_c}\left( x \right)$ is the potential clique and $Cl$ is the set of all possible cliques. When we extract the prior energy from potential clique, it gives us features that have similar characteristics as the clique, therefore we applied it over the static patterned signals. Figure 7 shows the results of prior energy function applied to each EM iteration, where red represents typing and blue shows resting motion patterns.

### Features optimization

#### Features optimization using quadratic discriminant analysis

**Figure 8:**
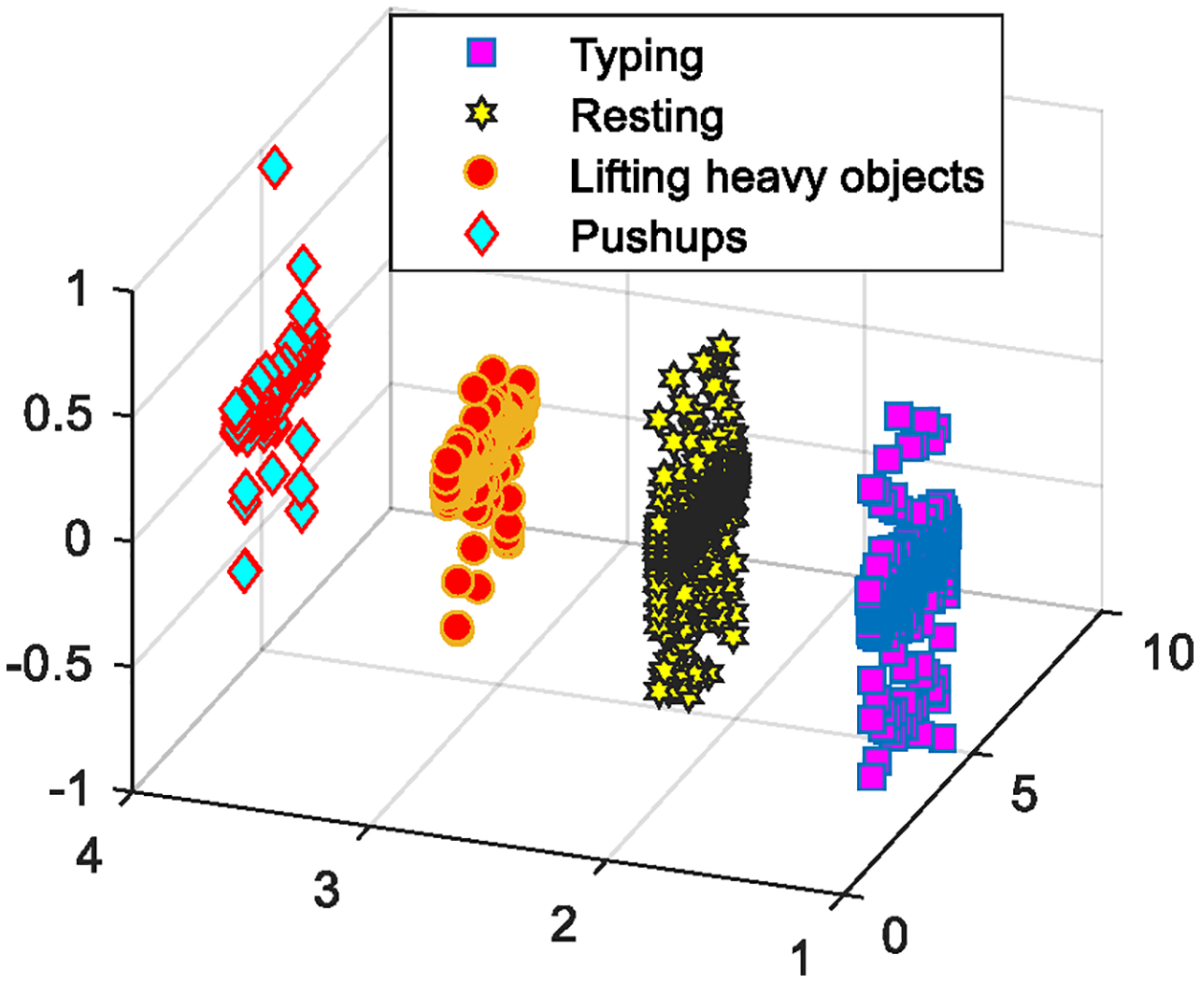
QDA based optimized features for kinematic and static activities.

The features extracted for both kinematic and static patterned signals are non-linear. Therefore, applying quadratic discriminant analysis (QDA) is suggested instead of linear techniques. QDA is used when it is not possible to assume the activity dispersion (Bose et al., 2015). So, we calculate covariance matrix ${\mu _m}$ for each activity $m\; \in \left\{ {1, \ldots ,\; M} \right\}$. QDA can be calculated as mentioned in Eq. (13):

(13) $${\delta _m}\left( x \right) = \; - \displaystyle{1 \over 2}\log \;\left| {\sum m} \right| - \; \displaystyle{1 \over 2}{(x - \; {\mu _m})^T}\mathop \sum \nolimits_m^{ - 1} \left( {x - {\mu _m}} \right) + log{\pi _m}$$

where ${\pi _m}$ represents $M$ activity priors and $x$ is the extracted features vector. Figure 8 shows the results of QDA applied on extracted features for both kinematic and static activities.

#### Feature optimization using orthogonal fuzzy neighborhood discriminant analysis

**Figure 9:**
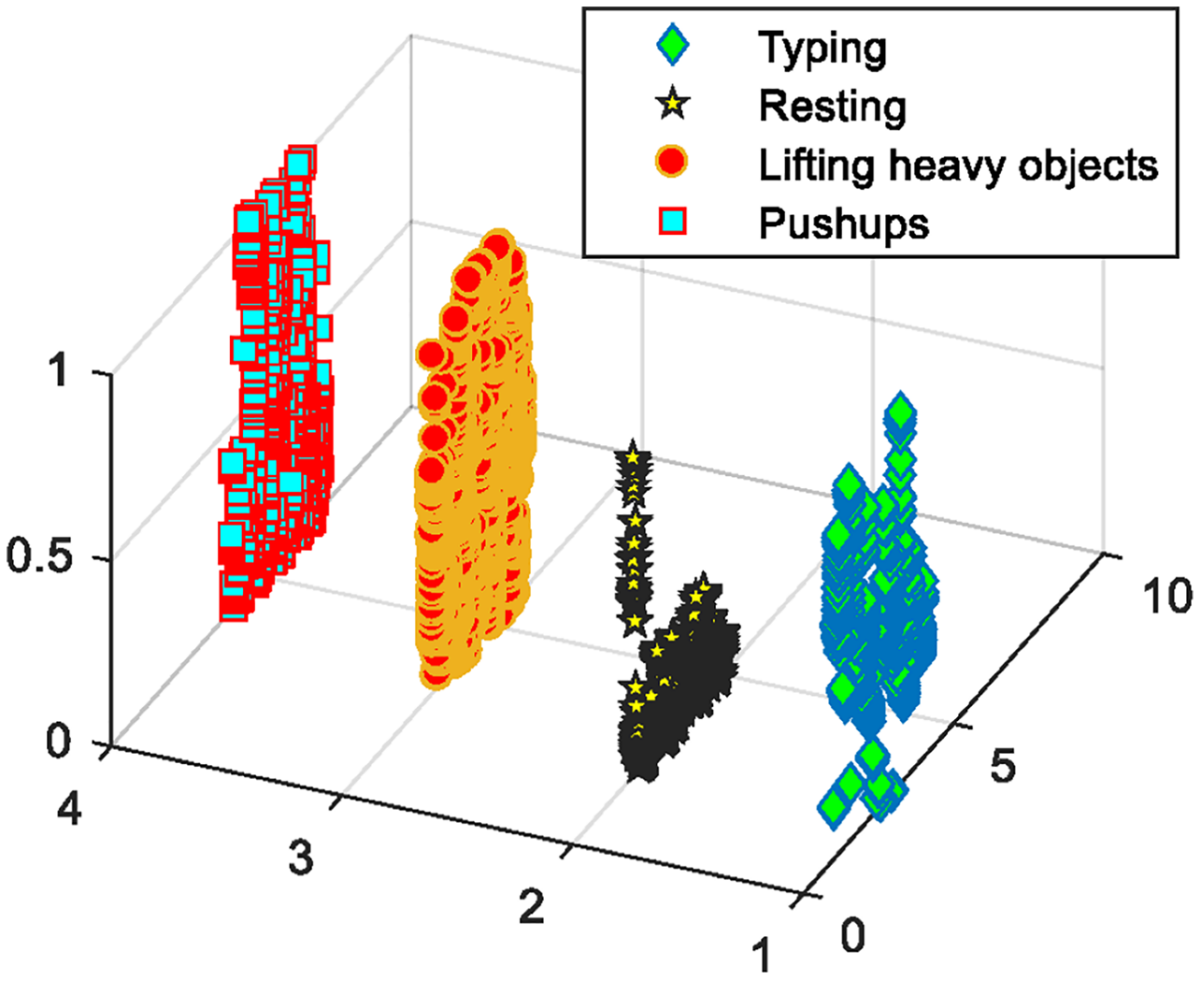
OFNDA based selected optimized features.

To maximize the distance between center of different classes and minimize the distance within the classes (Phukpattaranont et al., 2018), OFNDA is used as PHM model’s second features optimization technique. OFNDA also takes care of the contribution of the samples to the different activities by providing an orthogonal projection matrix (Khushaba, Al-Ani & Al-Jumaily, 2010). Equation (14) shows the proposed regularized objective function to determine the fuzzy partition matrix $F$ for grouping a collection of $d$ samples into $a$ activities:

(14) $${F_p}\left( {F,\; v} \right) = {\sum}_{k = 1}^d {\sum}_{i = 1}^a \mu _{ik}^p{\rm exp}{(\displaystyle{{\left| {{x_k} - {v_i}} \right|} \over {{\raise0.7ex\hbox{${{\eta _i}}$} \!\mathord{\left/ {\vphantom {{{\eta _i}} 3}}\right.} \!\lower0.7ex\hbox{$3$}}}})^2} - \lambda {\sum}_{i = 1}^a {\sum}_{k = 1}^d \left( {{\mu _{ik}} - 1} \right)$$

where ${\mu _{ik}}$ shows the membership grade of ${k^{th}}$ sample in the ${i^{th}}$ activity, $\lambda$ gives the language multiplier, $p$ is the fuzzification parameter, the means of input is ${v_i}$ from activity $i$, and ${\eta _i}$ represents the chosen radius for each activity as $max\left| {{x_k} - {v_i}} \right|$ having $k = 1,2, \ldots ,\; d$. Figure 9 demonstrates the OFNDA based selected features subset for PAD dataset.

### Classification using manifold regularization

The optimized extracted features are provided to the manifold regularization algorithms as input vectors. It is a family of algorithms based on regularization, which is beneficial in exploiting the geometry of marginal distribution. It can utilize supervised, semi-supervised, and unsupervised learnings of the labeled and unlabeled data.

Since our vectors are labeled, we used a semi-supervised learning technique to help in improving the learning curve by considering both types of data. As a result, PHM model is able to obtain natural out-of-sample extension for the new samples that are not learned by the classifier yet.

Manifold regularization methods are used to find a smooth low-dimensional manifold embedded in a high-dimensional vectors based on the signal data (Li et al., 2019). Eq. (15) shows the formula for calculating the regularized least squares (RLS) for manifold regularization:

(15) $${\hat f_\gamma } = arg \mathop {min }\limits_{f{\varepsilon} {\rm \; {\cal H}}} {\sum}_{i = 1}^n l\left( {{y_i},f\left( {{x_i}} \right)} \right) + {\gamma _A}|\left| f \right||_{\rm {\cal H}}^2 + {\gamma _I}{{f}^T}{Lf}$$

where ${\rm {\cal H}}$ is the reproducing kernal Hilbert space for functions $f$, $l$ is the loss function, ${L\; }$shows the Laplacian matrix, ${\gamma _A}$ tells the complexity of function in the ambient space and ${\gamma _I}$ is the complexity of function in the intrinsic space. Furthermore, the regularization problem algorithm becomes Laplacian regularized least squares (LapRLS) with squared loss function as shown in Eq. (16):

(16) $${\hat f_\gamma } = arg \mathop {min }\limits_{f\varepsilon {\rm \; {\cal H}}} {\sum}_{i = 1}^n {\left( {{y_i} - f\left( {{x_i}} \right)} \right)^2} + {\gamma _A}|\left| f \right||_{\rm {\cal H}}^2 + {\gamma _I}{{f}^T}{Lf}$$

Another algorithm called Nyström-PCG has been suggested by Daas, Rees & Scott (2021), which consists of two steps. First, Nyström uniform subsampling is done using the previous matrix from LapRLS and secondly, preconditioning has been introduced to accelerate the solution reducing the time to $O\left( {{n^{1.5}}} \right)$.

## Datasets description

The PAD dataset has been collected *via* surface electromyography (sEMG) and IMU sensors from 40 participants with equal gender distribution. It has been created to monitor the human muscles activity during routine activities including resting, typing, push up exercise, and lifting heavy objects. Total time for collection of each participant data is 70 s.

The GOTOV dataset is based on the healthcare monitoring issues for 35 elderly participants using multiple sensors, namely, accelerometer, human’s physical information sensors like heart rate. It consists of 3,400 s data for each participant. The dataset consists of sixteen activities including jumping, standing, step, lying down left, lying down right, sitting sofa, sitting couch, sitting chair, walking stairs up, washing dishes, stacking shelves, vacuum cleaning, walking slow, walking normal, walking fast, and cycling. If we take a closer look at the activities, we can see that both the datasets have multiple indoor-outdoor activities that will help in making a robustly performing PHM model.

## Results

**Experiment I:** Manifold regularization over datasets

**Figure 10:**
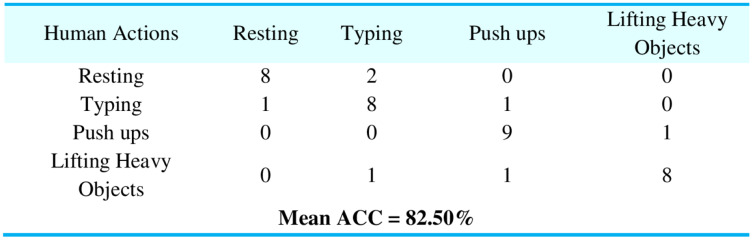
Confusion matrix results using manifold regularization over the PAD dataset.

**Figure 11:**
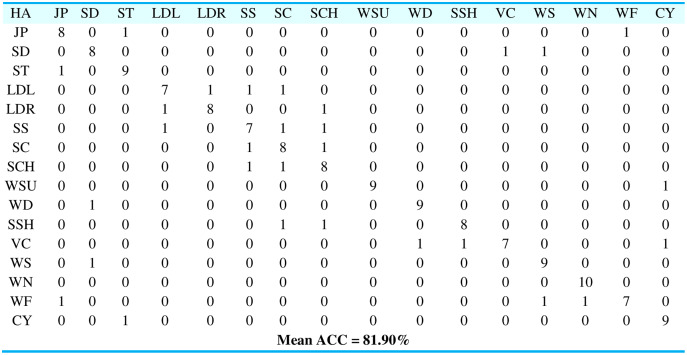
Confusion matrix results using manifold regularization over the GOTOV dataset.

To evaluate the performance of our proposed PHM model, we proposed the manifold regularization using RLS, LapRLS, and Nyström LapRLS algorithms. Figure 10 represents the results in the shape of the confusion matrix over PAD dataset providing the mean accuracy as 82.50%. Accuracy is one of the vital implications of our system because it shows how accurately the proposed method was able to detect the human motion patterns. Figure 11 represents the outcomes using confusion matrix over GOTOV dataset achieving the mean accuracy as 81.90%, where the accuracies are calculated using Eq. (17):

(17) $$ ACC = \; \displaystyle{{correctly\; classified\; activities} \over {all\; activities}}$$

HA = Human actions, JP = Jumping, SD = Standing, ST = Step, LDL = Lying down left, LDR = Lying down right, SS = Sitting sofa, SC = Sitting couch, SCH = Sitting chair, WSU = Walking stairs up, WD = Washing dishes, SSH = Stacking shelves, VC = Vacuum cleaning, WS = Walking slow, WN = Walking normal, WF = Walking fast, CY = Cycling.

**Experiment II:** RMSE *via* QDA features

**Figure 12:**
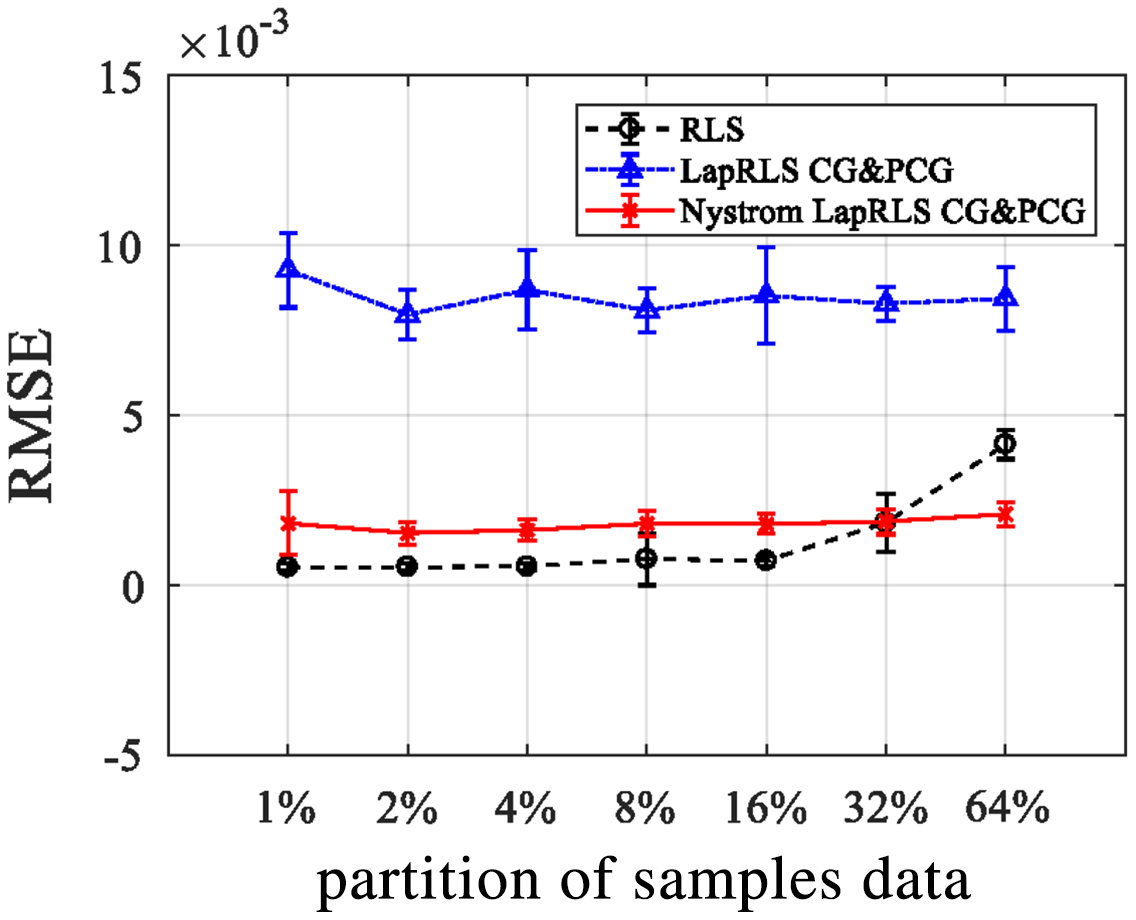
RMSE for RLS, LapRLS, and Nyström LapRLS over QDA features for PAD dataset.

For optimized features *via* QDA, we processed for the three manifold regularization algorithms and results are described in the form of root mean squared error (RMSE) when applied over different partitions of labeled and unlabeled data for semi-supervised learning as represented in Fig. 12. RMSE points out the standard deviation of prediction errors, therefore, it is observed that RLS has provided better results when influenced by the sample proportions over QDA features for both selected datasets. So, as the sample percentage increases, the RMSE decreases.

**Experiment III:** RMSE *via* OFNDA features

**Figure 13:**
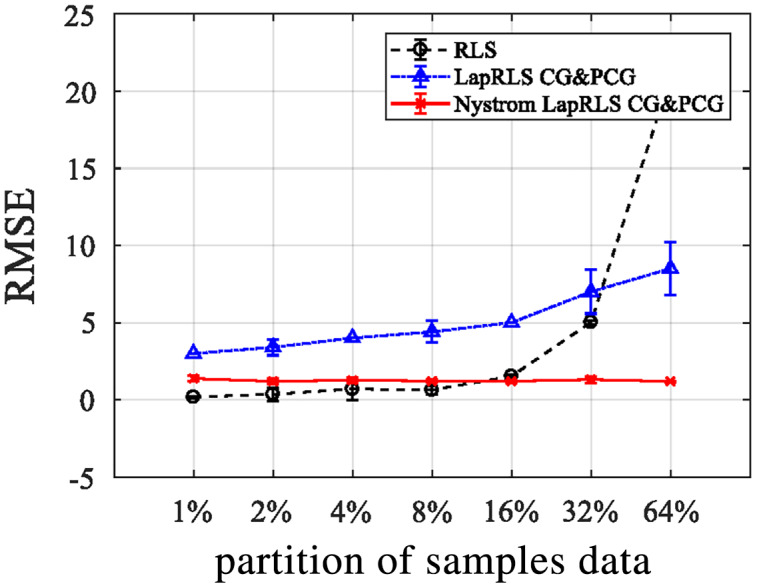
RMSE for RLS, LapRLS, and Nyström LapRLS over OFNDA features for PAD dataset.

All three algorithms of manifold regularization are applied over OFNDA features and we get results in the form of RMSE when applied different partitions of labeled and unlabeled data for semi-supervised learning as shown in the Fig. 13. Here, RLS delivers best outcomes when proportion of samples is low over OFNDA features for PAD and GOTOV datasets. So, when sample percentage decreases, RMSE also decreases.

**Experiment IV:** Comprehensive analysis over selected datasets

The experiment is performed over both PAD and GOTOV datasets to measure the results in the form of precision, recall, and F1-scores. Equations (18)–(20) represent the formulas for calculating these parameters. Table 2 shows the outcomes for PAD dataset, whereas Table 3 displays the results over the GOTOV dataset. The bold entries in both tables represents the mean values of precision, recall, and F1-score.

**Table 2:** Precision, recall, and F1-score results over PAD dataset.

Actions	Precision	Recall	F1-score
Resting	0.80	0.89	0.84
Typing	0.89	0.72	0.79
Push ups	0.90	0.81	0.84
Lifting heavy objects	0.80	0.89	0.84
**Mean**	**0.85**	**0.83**	**0.83**

**Table 3:** Precision, recall, and F1-score results over GOTOV dataset.

**Actions**	**Precision**	**Recall**	**F1-score**
JP	0.80	0.80	0.80
SD	0.80	0.80	0.80
ST	0.90	0.81	0.85
LDL	0.70	0.78	0.74
LDR	0.80	0.89	0.84
SS	0.70	0.70	0.70
SC	0.80	0.95	0.87
SCH	0.80	0.95	0.87
WSU	0.90	1.00	0.95
WD	0.90	0.90	0.90
SSH	0.80	0.89	0.84
VC	0.70	1.00	0.82
WS	0.90	0.81	0.85
WN	1.00	0.83	0.91
WF	0.70	0.87	0.77
CY	0.90	0.81	0.85
**Mean**	**0.82**	**0.86**	**0.83**

(18) $$Precision = \; \displaystyle{{true\; positives} \over {true\; positives + false\; positives}}$$

(19) $$Recall = \; \displaystyle{{true\; positives} \over {true\; positives + false\; negatives}}$$

(20) $$F1 - Score = \; \displaystyle{{2*Precision*Recall} \over {Precision + Recall}}$$

Precision over both datasets explain that our proposed PHM model is good at predicting the motion patterns. Similarly, recall suggests how many times our PHM model was correctly able to predict human actions. F1-score is extracted by combining both precision and recall performance metrics, therefore it describes the properties of both.

JP = Jumping, SD = Standing, ST = Step, LDL = Lying down left, LDR = Lying down right, SS = Sitting sofa, SC = Sitting couch, SCH = Sitting chair, WSU= Walking stairs up, WD = Washing dishes, SSH = Stacking shelves, VC = Vacuum cleaning, WS = Walking slow, WN = Walking normal, WF = Walking fast, CY = Cycling.

**Experiment V:** Comparison with other state-of-the-art PHM models

In Wang et al. (2018), transfer learning is utilized to save the cost and computational time. They proposed a stratified transfer learning framework to learn cross-domain activity recognition. First, majority voting is used to obtain pseudo labels, then it transfers both domains into the same. Average accuracy achieved for activity recognition is 61.37%. Xia et al. (2021) proposed a novel deep learning model for human motion prediction. They have contributed by proposing a big synthetic dataset using IMU and multi-level domain adaptive learning. However, the system was able to achieve a mean accuracy rate of 73.6%. Lawal & Bano (2019) proposed an automated monitoring system for elderly health issues. They have utilized waist mounted sensors and CNN to train for the motion signals. For both static and dynamic activities, the system was able to achieve 78.0% accuracy.

Al-Naser et al. (2018) proposed a hierarchical framework that learns and classifies unidentified activities. Object recognition module, myo-armband, and activity recognition have been utilized to perform the complex activity detection and achieved 77.0% precision along with 82.0% recall rates. Kwon, Abowd & Plotz (2021) presented a body-worn model based on virtual and real IMUs. Essential information from the IMUs is extracted and presented a maximum of 80.2% accuracy rate. Paraschiakos et al. (2020) introduced an algorithm called LARA for tracking elderly motion using combinations of wearable sensors. They have considered the granularity of each action using prior biological knowledge. In two-body locations, they could achieve a maximum of 81.0% accuracy for chest-wrist-equivital sensors combination.

Kwon et al. (2021) have used the IMUTube concept for human action recognition method. They analyzed free-weight gym exercises having a range of artifacts like video noise, non-human poses, body parts occlusions *etc*. The IMUTube system was able to achieve a maximum of 81.5% recognition accuracy. Table 4 presents the mean accuracy comparison of PHM model with other state-of-the-art systems that shows the PHM model’s efficiency and effectiveness in bold over the other methods.

**Table 4:** Motion prediction mean accuracy comparison with other PHM methods.

PHM methods	Accuracy (%)
Wang et al. (2018)	61.37
Xia et al. (2021)	73.61
Lawal & Bano (2019)	78.00
Al-Naser et al. (2018)	80.00
Kwon, Abowd & Plotz (2021)	80.20
Paraschiakos et al. (2020)	81.00
Kwon et al. (2021)	81.50
**Proposed PHM method**	**82.20**

## Conclusion and future work

The proposed PHM model is based on noise reduction, data windowing, patterns recognition, features extraction, and optimization techniques for feature dimensionality reduction. To evalaute the performance of the proposed model, two benchmarked datasets, namely, PAD and GOTOV, were used. For noise reduction, a state-of-the-art filter was proposed for IMU signals and a Bessel filter was utilized for the physiological sensory data. For-pre-classification, patterns were decided on the basis of polynomial probability distribution. Kinematic and static motion patterns were separated and fed to the features extraction techniques. Two optimization procedures were suggested for PHM model including QDA and OFNDA. Accuracy rates of 82.50% and 81.90% have been achieved for PAD and GOTOV datasets, respectively. PHM model has shown a great improvement when compared to the existing systems in literaure.

The drawback is that motion complexity of different patterns causes restricted decision-making for patterns identification. The selected algorithms were not able to detect extremely complex patterns. Another limitation is the size of datasets used. In future, this work can be applied to larger datasets with diverse range of activities in order to predict additional motion patterns.

## Supplemental Information

10.7717/peerj-cs.1105/supp-1 Supplemental Information 1 Code for pre-processing.

10.7717/peerj-cs.1105/supp-2 Supplemental Information 2 Optimization Code.

10.7717/peerj-cs.1105/supp-3 Supplemental Information 3 Raw Dataset.

10.7717/peerj-cs.1105/supp-4 Supplemental Information 4 Details of time along with x, y, and z-axes of the gyroscope readings.

10.7717/peerj-cs.1105/supp-5 Supplemental Information 5 Details of time along with x, y, and z-axes of the accelerometer readings.

## Additional Information and Declarations

### Data Availability

The following information was supplied regarding data availability: The raw data is available in the Supplemental Files. The PAD Dataset is available at Mendeley: Khan, Asad Mansoor; Khawaja, Sajid Gul; Akram, Muhammad Usman; Khan, Ali Saeed (2020), “Physical Action Dataset ”, Mendeley Data, V2, DOI 10.17632/bcv9vsxkyc.2. The GOTOV Dataset is available at: Paraschiakos, Stylianos; Beekman M. (Marian); Knobbe A. (Arno); Cachucho R. (Ricardo); Slagboom P. (Eline) (2020): GOTOV Human Physical Activity and Energy Expenditure Dataset on Older Individuals. 4TU.ResearchData. Dataset. DOI 10.4121/12716081.v2.
